# Late-Onset Fabry Disease Affecting the Kidneys and Liver While Sparing the Heart: A Case Report

**DOI:** 10.7759/cureus.30989

**Published:** 2022-11-01

**Authors:** Qusai Alitter

**Affiliations:** 1 Pulmonary Disease, Larkin Community Hospital Palm Springs Campus, Miami, USA

**Keywords:** late-onset fabry disease, liver disease, proteinuria, chronic kidney disease (ckd), cardiomyopathy, left ventricular hypertrophy, cardiac conduction disorder, echocardiography - heart failure - valvular heart disease

## Abstract

Fabry disease (FD), also known as Anderson-Fabry disease, is an X-linked inherited lysosomal storage disorder caused by the deficiency or reduced activity of alpha-galactosidase A enzyme, which results in the accumulation of globotriaosylceramide (Gb3) in the cells. Atypical (late-onset) FD is characterized by the preserved residual activity of alpha-galactosidase A enzyme resulting in a later presentation in life than classic FD. Patients with late-onset FD are usually present in their third to seventh decades of life with the heart being the most commonly affected organ. FD can also affect the renal and gastrointestinal (GI) systems, however, in the literature, FD limited to the kidneys is scarcely reported and there is no data to suggest disease involvement of the liver. We present a rare case of late-onset FD affecting the kidneys and liver without cardiac or other organ involvement in a patient without having a family history of FD.

## Introduction

Fabry disease (FD) affects all ethnic and racial groups in an X-linked mode of inheritance [1]. Late-onset FD affects individuals in their third to seventh decades of life with an estimated prevalence of 1:1000 to 1:3000 in males and 1:6000 to 1:40,000 in females [1]. However, the prevalence of FD is believed to be underestimated due to the challenges in reaching the diagnosis that are attributed to the nonspecific clinical features and varying presentations of the disease.

FD can have a multi-systemic involvement leading to cardiac, cutaneous, ocular, neurological, renal, and/or gastrointestinal (GI) manifestations with the cardiac variant being the most common variant of late-onset FD presenting with left ventricular hypertrophy (LVH) [2-4], valvular and conduction abnormalities [5-7], and/or coronary artery disease (CAD) [8]. Angiokeratoma occurs in 66% of male and 36% of female patients with FD and is usually linked to renal involvement [9,10]. Ocular involvement is not uncommon in patients with FD with corneal opacities (cornea verticillata) and conjunctival vessel tortuosity being characteristic features of the disease [11]. Neurological manifestations of the disease can include cerebrovascular accidents, hearing loss, and/or acroparesthesia [12]. FD can also affect the gastrointestinal (GI) system with diarrhea and abdominal cramping being the most commonly reported symptoms by affected individuals [13,14], however, in the literature review, there is no evidence to suggest liver involvement. The renal variant of the disease most commonly presents with proteinuria, chronic kidney disease (CKD), and/or end-stage kidney disease (ESKD) with a prevalence of 20% in patients with CKD [15], however, FD limited to the kidneys is scarcely reported in the literature [16].

We present a case of a patient with elevated kidney and liver functions secondary to late-onset FD without evidence of cardiac or other organ involvement.

## Case presentation

**Figure 1:**
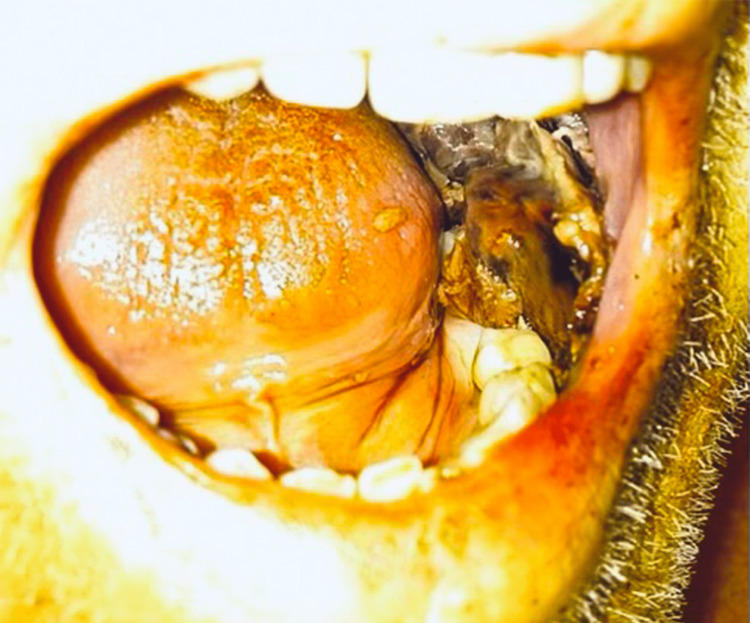
Oral cavity and buccal mucosa Bilateral black buccal mucosal ulceration

A 68-year-old male with a past medical history of essential hypertension, dyslipidemia, chronic obstructive pulmonary disease, nicotine dependence, complicated diverticulitis with subsequent partial sigmoid colectomy, and obesity presented to the hospital with anuria for four days. Physical examination was significant for bilateral black buccal mucosal ulceration (Figure 1) and +1 pitting edema of the lower extremities. Blood work was significant for electrolyte imbalance, elevated renal and liver functions, metabolic acidosis, elevated C-reactive protein, and negative autoimmune and viral hepatitis panel (Table 1).

**Table 1:** Blood Tests and Diagnostic Workup Blood work was significant for electrolyte imbalance, elevated renal and liver functions, metabolic acidosis, elevated C-reactive protein, and negative autoimmune and viral hepatitis panel.

Blood Tests and Diagnostic Workup	Result	Reference Range
White Blood Cell Count	9.6 K/mcL	4.00-10.80 K/mcL
Hemoglobin	15.3 g/dl	14-17 g/dl
Hematocrit	43.7 %	42-52%
Platelet Count	160 K/mcL	130-400 K/mcL
Sodium	125 mmol/L	137-146 mmol/L
Potassium	5.3 mg/dl	3.6-5 mmol/L
Carbon Dioxide	16 mmol/L	21-32 mmol/L
Chloride	92 mmol/L	98-112 mmol/L
Blood Urea Nitrogen	82 mg/dl	8-25 mg/dl
Serum Creatinine	11 mg/dl	0.70-1.20 mg/dl
Alanine Transaminase	110 U/L	12-64 U/L
Aspartate Aminotransferase	179 U/L	9-40 U/L
Total Bilirubin	1.2 mg/dl	0.2-1.1 mg/dl
Alkaline Phosphatase	333 U/L	38-127 U/L
C-Reactive Protein	17.10 mg/dl	<0.8 mg/dl
Erythrocyte Sedimentation Rate	10 mm/hr	<15 mm/hr
C3 Complement	127 mg/dl	79-152 mg/dl
C4 Complement	34.4 mg/dl	16-38 mg/dl
Anti-nuclear Antibodies	Negative	Negative
Anti-Smooth M*uscle* Antibodies	Negative	Negative
Anti-Myeloperoxidase Antibody	Negative	Negative
Anti-Protease 3 Antibody	Negative	Negative
Hepatitis A Virus IgM Antibody	Negative	Negative
Hepatitis B Virus Surface Antigen	Negative	Negative
Hepatitis B Virus Core Antibody	Negative	Negative
Hepatitis C Virus Antibody	Negative	Negative
Serum Cryoglobulin	Not Detected	Not Detected
Iron	31 mcg/dl	65-175 mcg/dl
Total Iron Binding Capacity	184 mcg/dl	250-450 mcg/dl
Transferrin Saturation	17%	20-50%
Ferritin	785 ng/ml	26-388 ng/ml

**Figure 2:**
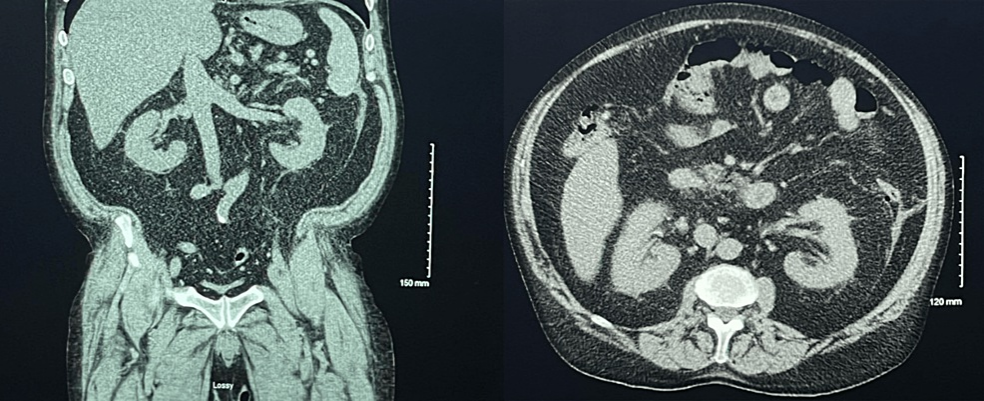
CT scan of the abdomen and pelvis Computed tomography (CT) scan of the abdomen and pelvis demonstrated normal morphology of the kidneys and liver. Left-hand side image: Coronal view, Right-hand side image: Axial view

A computed tomography scan of the abdomen and pelvis revealed normal kidneys and liver morphology and post-operative changes reflecting a history of partial sigmoid colectomy (Figure 2). The right upper quadrant ultrasound of the liver was unremarkable. The echocardiogram reported preserved ejection fraction without evidence of left ventricular hypertrophy or significant valvular abnormality.

Ear, nose, and throat service performed a biopsy of the buccal lesions which revealed hemorrhage and fibrin deposits with a benign and reactive squamous mucosa without evidence of a neoplastic or malignant process. Nephrology service felt elevated kidney function and electrolytes imbalance are due to volume depletion and started patient on intravenous fluids however, kidney function did not improve, therefore dialysis was initiated and a kidney biopsy was performed. A biopsy of the kidney revealed acute tubular necrosis with 15% interstitial fibrosis and some myoglobin casts. Zebra bodies within the glomerular podocytes were also noted. The diagnosis of FD was entertained, and leukocyte alpha-galactosidase A level was found to be reduced along with an elevated serum level of globotriaosylsphingosine confirming the diagnosis of late-onset FD. 

## Discussion

FD is a rare X-linked inherited disorder with multiple variants and a wide range of clinical presentations making it difficult to diagnose. The heart is the most common organ to be affected and is almost always involved in late-onset FD presenting with LVH [2-4], valvular and conduction abnormalities [5-7], and CAD [8]. The kidney variant of the disease is believed to be underestimated and scarcely reported in the literature [16]. It presents most commonly with proteinuria, hypertension, CKD, and/or ESKD and is estimated to be responsible for 20% of CKD cases [15]. GI system can also be involved in FD with diarrhea and abdominal cramping being the most commonly reported symptoms by affected individuals [13,14], however, there is no data in the literature to suggest liver involvement. FD patients tend to have other clinical features suggestive of the disease including angiokeratoma, acroparesthesias, hypohidrosis, hearing loss, transient ischemic attack (TIA), cerebrovascular accident (CVA), and/or corneal opacities.

Our case is unique in that this patient only demonstrated renal and liver dysfunction while other reported cases of late-onset FD presented mainly with cardiomyopathy and other clinical features of the disease. Our patient presented with a progressive decline of kidney function requiring dialysis and elevated liver function tests without a clear etiology and had an unremarkable echocardiogram. Furthermore, the patient did not express the common clinical manifestations of the disease mentioned earlier. To our knowledge, this is the first case of atypical FD involving the kidneys and liver without affecting the heart or other organs. 

FD patients tend to be misdiagnosed or experience a delay in diagnosis due to the rarity, nonspecific clinical symptoms, and varying presentations of the disease. Obtaining a detailed patient’s personal and family history that targets the clinical manifestations of the disease along with performing a meticulous physical examination and work-up can be good preliminary guidance to its diagnosis. Treatment options include enzyme replacement therapy for qualified patients, pain management with anticonvulsants, analgesics, non-steroidal anti-inflammatory medications, supportive care with antihypertensives, antiarrhythmics, and dialysis for patients with ESKD.

## Conclusions

This case highlights the challenges in diagnosing FD due to the rarity, nonspecific manifestations, and varying presentations of the disease. It also emphasizes the lack of common clinical features or the absence of cardiac manifestations of FD should not exclude the diagnosis. FD should be suspected in patients with a progressive decline of kidney function and elevated liver function tests without a clear etiology. Obtaining a detailed personal and family history of the patient along with performing a thorough physical examination and workup are crucial steps in preventing diagnostic delays and the early initiation of treatment in patients with FD. 
